# 

**Published:** 1891-09

**Authors:** 


### HALL'S JOURNAL of HEALTH

ESTABLISHED 1854

CONTENTS.

Vol. 38 No. 8. 9

| | PAGE. |
|---|---|
| A Word with our Readers.. .. | 193 |
| Curiosities of the Vegetable Kingdom ......... ....... | 194 |
| A Monster to be avoided...... | 197 |
| The Age of Decay ...... ..... | 198 |
| Under the Earth ... .......... | 199 |
| Cancer . .................... | 200 |
| What is a Cell ?........ ...... | 201 |
| Sponging out a Headache ... . | 202 |
| A Narrow Chance............ | 203 |
| Worth Remembering... ....... | 204 |
| Water as a Medicine .... ... | 205 |
| A Case of Trance ..... ...... | 206 |
| Worms .... ............ ...... | 207 |
| All Forms of Life Cellular .... | 208 |
| Hot Milk a Stimulant ........ | 208 |
| Victor Hugo's Presage of Immortality ................. | 209 |
| How to Avoid Choking . ... | 210 |
| Neatness in Dress at Home.... | 210 |
| Aluminum ................... | 211 |
| Miscellaneous ............ .. . | 212 |
| Celery ...... ... ........... | 212 |
| Speed of the Electric Current . | 213 |
| The First Meerchaum Pipe.... | 213 |
| To Take Out Paint ........... | 215 |
| Literary ...................... | 216 |
| The Retort.............. . ... | 216 |

INDEX TO ADVERTISEMENTS OF HALF A PAGE AND OVER.

| | PAGE. |
|---|---|
| Horsford's Acid Phosphate... | I |
| Lydia E. Pinkham Med. Co.. | II |
| Rochester Lamp Co......... .. | III |
| Erie Medical Co.............. | IV |
| Ruckel & Hendel....... ..... | V |
| Dr. J. C. Ayer & Co ......... | V |
| Kahn Tailoring Co. .... ...... | VIII |
| Herba Vita.................... | VIII |
| Hall's Journal Premiums. ... | IX |
| Hall's Journal of Health ...... | X |
| Royal Baking Powder........ | XI |
| I. S. Johnson & Co........... | XI |

